# Clonal hematopoiesis of indeterminate potential and the risk of breast cancer: a UK Biobank cohort study

**DOI:** 10.1097/JS9.0000000000003060

**Published:** 2025-06-17

**Authors:** Jiayi Lai, Zhihui Xi, Dandan Zhu, Qiyu Zhang, Yifei Yang, Xin Wang, Baijin Xia, Tieying Hou, Huolun Feng

**Affiliations:** a School of Medical and Information Engineering, Gannan Medical University, Ganzhou, China; b Department of Gastrointestinal Surgery, Department of General Surgery, Guangdong Provincial People’s Hospital (Guangdong Academy of Medical Sciences), Southern Medical University, Guangzhou, Guangdong, China; c School of Medicine, South China University of Technology, Guangzhou, Guangdong, China; d Guangdong Center for Clinical Laboratory, Guangdong Provincial People’s Hospital (Guangdong Academy of Medical Sciences), Southern Medical University, Guangzhou, Guangdong, China; e The First Clinical Medical School, Southern Medical University, Guangzhou, Guangdong, China; f Guangdong Cardiovascular Institute, Guangdong Provincial People’s Hospital (Guangdong Academy of Medical Sciences), Guangzhou, Guangdong, China; g Medical Research Institute, Guangdong Provincial People’s Hospital (Guangdong Academy of Medical Sciences), Southern Medical University, Guangzhou, Guangdong, China; h Medical Experimental Center, Shenzhen Nanshan People’s Hospital, Shenzhen, Guangdong, China; i Medical School, Shenzhen University, Shenzhen, Guangdong, China

**Keywords:** breast cancer, clonal hematopoiesis of indeterminate potential, risk factor, UK Biobank

## Abstract

**Objective:**

This study aims to investigate the association between clonal hematopoiesis of indeterminate potential (CHIP) and the risk of breast cancer.

**Methods:**

We analyzed data from the UK Biobank, initially comprised 502 170 participants who met our inclusion criteria. Baseline data on age, body mass index (BMI), alcohol consumption, smoking habits, diet quality scores, physical activity levels, and health status. Whole-exome sequencing data were used to detect CHIP. Logistic regression and Cox proportional hazards (CoxPH) models were employed to assess the association between CHIP and breast cancer risk, adjusting for potential confounders. Additionally, we compared cumulative incident breast cancer cases between CHIP and non-CHIP groups.

**Results:**

Univariable analysis showed that CHIP had a higher risk of developing breast cancer (odds ratio [OR] (95% confidence interval [CI]) = 1.200 [1.09–1.31], *P* = 8.94e-05). This association persisted after adjusting for potential confounders related to lifestyle, inflammation, health status and genetic factors (OR [95% CI] = 1.19 [1.09–1.31], *P* = 1.77e-04). The correlation remained consistent even after excluding participants with extreme BMI or those diagnosed with breast cancer within 2 or 5 years of follow-up (all OR = 1.18, *P* < 0.01). Additionally, individuals with CHIP exhibited a significantly higher incidence of breast cancer compared to non-CHIP individuals (Log-Rank *P* = 5.19e-05). The risk of breast cancer associated with CHIP was predominantly driven by mutations in the gene *ATM* (OR [95% CI] = 3.97 [2.89–5.36], *P* = 2.02e-18), and *DNMT3A* (OR [95% CI] = 1.33 [1.03–1.66], *P* = 0.014).

**Conclusions:**

CHIP is associated with an increased risk of breast cancer, particularly influenced by the *ATM* and *DNMT3A* genes.

## Introduction

Breast cancer is a significant health concern among women and a leading cause of cancer-related deaths, ranking as the second most diagnosed cancer globally^[1]^. Both primary prevention and secondary prevention strategies can reduce breast cancer morbidity and mortality^[2]^. Early detection of breast cancer is particularly crucial, as there is a 70–80% cure rate in breast cancer cases at early, nonmetastatic stage^[3]^. Various lifestyle and environmental factors, such as unhealthy diet, high alcohol consumption and lack of physical activity, contribute to the development of breast cancer^[2]^. Despite the identification of numerous risk factors^[4]^, discovering new risk factors could broaden the scope of high-risk populations for cancer screening, enabling earlier detection and improved outcomes.

Clonal hematopoiesis of indeterminate potential (CHIP) is characterized by the presence of mutations related to hematological neoplasms in blood or bone marrow, without meeting the diagnostic criteria for hematological malignancies or other clonal disorders^[5]^. Early epidemiological studies have shown that CHIP is associated with a significantly elevated risk of hematologic malignancies, with a hazard ratio (HR) of approximately 11 and a 40% increase in mortality^[6]^. Prevalence of CHIP rises with age and is commonly observed in older individuals^[7]^. About 80% of CHIP cases involve mutations in genes regulating epigenetic process, such as *DNMT3A, TET2, ASXL1*, DNA damage repair genes *PPM1D, TP53*, regulatory tyrosine kinase JAK2, and mRNA splicing components SF3B1 and SRSF2^[8]^, and these mutations could be detected through whole-exome or -genome sequencing (WES or WGS) on peripheral blood samples^[9]^.

CHIP is associated with dysregulated inflammatory responses in immune cells like macrophages, which may result from an altered microenvironment, disrupting normal inflammatory processes critical for immune function^[10,11]^. While CHIP has been linked to cardiovascular diseases (CVDs), such as coronary artery disease^[10,12]^ and arrhythmias^[13]^, recent studies also associate it with higher mortality rates in patients with solid tumors, including lung cancer^[14–16]^. In breast cancer, previous research found CHIP to be uncommon in young women with early-stage breast cancer after cytotoxic exposure^[17]^. A clinical trial (NCT03858322) involving elderly breast cancer patients treated with the ‘ADVANCE’ ADjuVANt chemotherapy observed varied CHIP dynamics including emergence, expansion, contraction, and disappearance^[18]^. Another study on a cohort of 234 patients with early breast cancer found that CHIP was frequent at baseline but there was no significant difference between patients receiving chemotherapy or not receiving it^[19]^. Despite these findings, the underlying relationship between CHIP and the risk of breast cancer remains largely unexplored.

This study leverages WES data from a large population cohort in the UK Biobank to examine the hypothesis that CHIP may contribute to an increased risk of breast cancer, aiming to provide valuable insights into high-risk populations and enhance early detection strategies on breast cancer. The work has been reported in line with the STROCSS criteria^[20]^ and TITAN criteria^[21]^.

## Methods

### Study cohort

This study utilized data from the UK Biobank under approved application number 98937. The UK Biobank is a comprehensive biomedical database containing extensive on over 500 000 participants recruited between 2006 and 2010 from across the UK, with regular follow-ups. Baseline and follow-up data included sociodemographic, lifestyle, and health information, along with collected biospecimens. Over time, additional data on metabolic^[22]^, proteomic^[23]^, and genetic^[24]^ profiles were made publicly available for research purposes.

### Data collection

Basic lifestyle characteristics of each participant including age, sex, body mass index (BMI), alcohol intake status, smoking status, and physical activity were obtained and analyzed on the website of UK Biobank research analysis platform (UKB-RAP). Disease records and cancer diagnosis during the follow-up were also extracted on UKB-RAP according to the International Classification of Diseases Tenth or Ninth Revision (ICD-10 or ICD-9) (Supplementary Digital content, SDC, Table 1, Available at: http://links.lww.com/JS9/E845). Additional data on inflammation, genomic information and diet quality score^[25]^ were derived from relevant records, as detailed in SDC, Table 2, Available at: http://links.lww.com/JS9/E845

**HIGHLIGHTS**

- **UK Biobank Cohort Study**: Investigated the link between clonal hematopoiesis of indeterminate potential (CHIP) and breast cancer risk.
- **Higher Breast Cancer Risk**: Individuals with CHIP showed a significantly elevated risk of developing breast cancer.
- **Persistent Association**: The correlation held after adjusting for lifestyle, inflammation, health status, and genetic factors.
- **Genetic Influence**: Risk associated with CHIP was significantly influenced by mutations in *ATM* and *DNMT3A* genes.
- **Incidence Comparison**: CHIP group exhibited a higher incidence of breast cancer compared to non-CHIP group.
- **Implications**: Findings suggest CHIP as a potential risk factor for breast cancer, warranting further research.

### Data filtration

**Figure 1.**
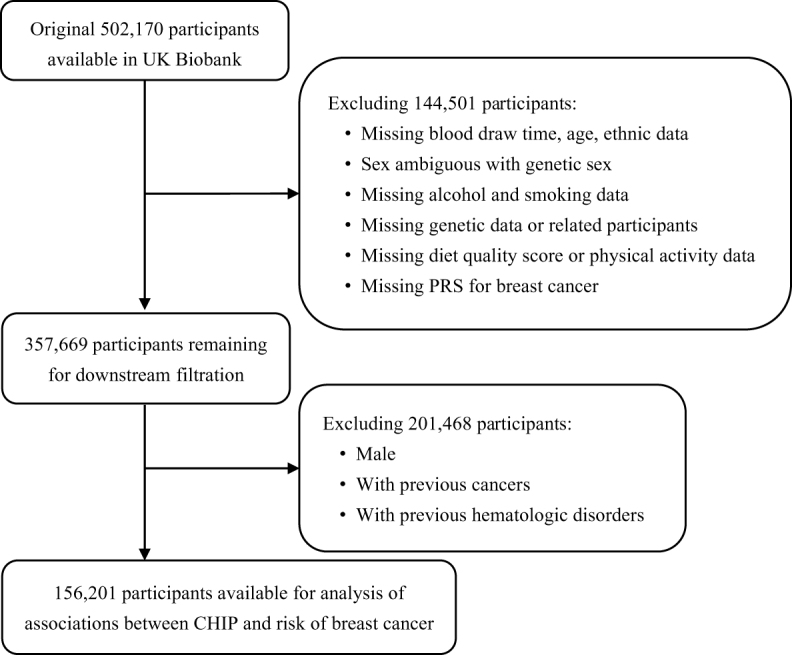
The schematic diagram illustrates the data filtration in the cohort from UK Biobank.

A sequential filtration process, illustrated in Figure 1, was employed to select the final cohort for analysis. Participants missing data on blood draw time, alcohol and smoking status, genetic data, diet quality scores, physical activity, or polygenic risk score (PRS) for breast cancer were excluded, resulting in a cohort of 357 669 participants. Further exclusions of males, individuals with prior cancer and those with hematologic disorders, left 156 201 participants for analyzing the associations between CHIP and risk of breast cancer.

### CHIP assessment

CHIP was assessed using CRAM files processed through the OQFE pipeline, as described in previous studies^[26]^, exclusively analyzed on UKB-RAP. CHIP-related gene mutations, identified from WES data of blood samples, included DNMT3A, TET2, ASXL1, among others listed in Supplementary Digital content, SDC, Table 3, Available at: http://links.lww.com/JS9/E845. Variant allele frequency (VAF) was calculated for each gene, categorizing participants into high VAF (VAF ≥ 10%) and low VAF (2% < VAF < 10%) groups according to the conventional and frequently used thresholds^[5,9,27]^.

### Exposures, confounders, and outcomes

Participants were classified into case (breast cancer diagnosis during follow-up) and control (no breast cancer diagnosis) groups, based on cancer diagnosis and blood draw dates. CHIP status at baseline was considered the exposure, with potential confounders grouped into four categories: (a) basic characteristics/lifestyle (race, age, BMI, alcohol consumption, smoking status, diet quality score, physical activity), (b) inflammation-related indices (C-reactive protein [CRP], systemic inflammation index [SII], pan-immune inflammation value [PIV], lymphocyte-to-monocyte ratio [LMR], neutrophil-to-lymphocyte ratio [NLR], platelet-to-lymphocyte ratio [PLR]), (c) genetic factors (first 10 components of genetic ancestry, standard PRS for breast cancer), and (d) health status/diseases (history of diabetes, high cholesterol, hypertension, chronic kidney disease, CVD).

### Statistical analysis

The association between CHIP status and breast cancer risk was examined using logistic regression and Cox proportional hazards (CoxPH) models. Multivariable analyses included grouped known risk factors as additive variables. Stratified analyses were performed to assess potential confounding by these factors. Sensitivity analyses, excluding participants with extreme BMI and those diagnosed within the first 2 or 5 years of follow-up, were conducted to minimize confounding effects. Odds ratios (OR) and HRs were used to indicate breast cancer risk, with statistical significance set at *P* < 0.05.

## Results

### Baseline characteristics of the cohort

The UK Biobank data underwent sequential filtration as depicted in Figure 1. The final cohort comprised 7727 breast cancer cases and 148 474 controls (Table 1). In the breast cancer group, the smoking prevalence (ever or current smokers) was 33.1%, higher than the control group’s 31.3%. Physical activity levels were lower in the breast cancer group (40.2 MET-h/week) compared to the controls (42.4 MET-h/week). Inflammatory markers were slightly elevated in the breast cancer group (CRP: 2.58 vs 2.49, SII: 604 vs 598, PIV: 271 vs 263). Furthermore, a higher prevalence of diabetes and hypertension was observed in breast cancer cases, along with an elevated PRS for breast cancer. Among the histology subtypes of breast cancer, “Infiltrating duct carcinoma, NOS” was predominant subtype (50.6%), followed by “Intraductal carcinoma, non-infiltrating, NOS” (10.9%) and “Lobular carcinoma” (9.65%). When stratified by CHIP status (Supplementary Digital content, SDC, Table 4, Available at: http://links.lww.com/JS9/E845), no significant baseline differences were found between CHIP and non-CHIP individuals, although CHIP individuals exhibited higher inflammation levels, consistent with breast cancer stratification.

**Table 1:** Baseline characteristics between controls and breast cancer cases

	Controls	Cases
	*N* = 148 474	*N* = 7727
Basic characteristics		
Age at blood draw, Mean ± SD	55.5 ± 8.04	56.2 ± 7.87
Race (white), *N* (%):	134 369 (90.5)	7063 (91.4)
BMI, kg/m^2^, Mean ± SD	26.8 ± 5.03	27.1 ± 4.93
Alcohol intake, *N* (%):		
Current	136 171 (91.7)	7130 (92.3)
Previous	4814 (3.24)	255 (3.30)
Never	7489 (5.04)	342 (4.43)
Smoking status, *N* (%):		
Current	12 284 (8.27)	641 (8.30)
Previous	46 472 (31.3)	2554 (33.1)
Never	89 718 (60.4)	4532 (58.7)
Diet quality score, Mean ± SD	54.6 ± 11.6	54.7 ± 11.5
Physical activity, MET-h/week, Mean ± SD	42.4 ± 40.9	40.2 ± 40.0
Inflammation		
CRP, Mean ± SD	2.49 ± 3.99	2.58 ± 3.95
SII, Mean ± SD	598 ± 337	604 ± 340
NLR, Mean ± SD	2.24 ± 1.07	2.26 ± 1.14
PLR, Mean ± SD	144 ± 59.0	144 ± 62.5
LMR, Mean ± SD	5.11 ± 3.33	5.04 ± 3.37
PIV, Mean SD	263 ± 273	271 ± 336
Health status		
History of diabetes, *N* (%):	1608 (1.08)	100 (1.29)
High cholesterol, *N* (%):	14 415 (9.71)	736 (9.53)
Hypertension, *N* (%):	7718 (5.20)	453 (5.86)
Chronic kidney disease, *N* (%):	67 (0.05)	4 (0.05)
Cardiovascular disease, *N* (%):	2998 (2.02)	155 (2.01)
Standard PRS of breast cancer, Mean ± SD	−0.18 ± 1.00	0.39 ± 1.00
Histology of breast cancer, *N* (%):		
Infiltrating duct carcinoma, NOS		3910 (50.6)
Intraductal carcinoma, noninfiltrating, NOS		845 (10.9)
Lobular carcinoma, NOS		746 (9.65)
Others		746 (9.65)
Missing		1480 (19.2)
CHIP status, *N* (%):		
No CHIP	139 972 (94.27)	7202 (93.21)
CHIP mutations, *N* (%):		
≥2 mutations	282 (0.19)	21 (0.27)
1 mutation	8220 (5.54)	504 (6.52)
CHIP VAF, *N* (%):		
≥10%	7633 (5.14)	482 (6.24)
2% to <10%	869 (0.59)	43 (0.55)

BMI, body mass index; CHIP, clonal hematopoiesis of indeterminate potential; CRP, C-reactive protein; LMR, cell count ratio of lymphocytes to monocytes; MET-h, metabolic equivalent in hour; *N*, numbers; NLR, cell count ratio of neutrophils to lymphocytes; NOS, not otherwise specified; PIV, pan-immune inflammation value; PLR, cell count ratio of platelet to lymphocytes; PRS, polygenic risk score; SII, systemic inflammation index.

### CHIP and breast cancer risk

**Figure 2.**
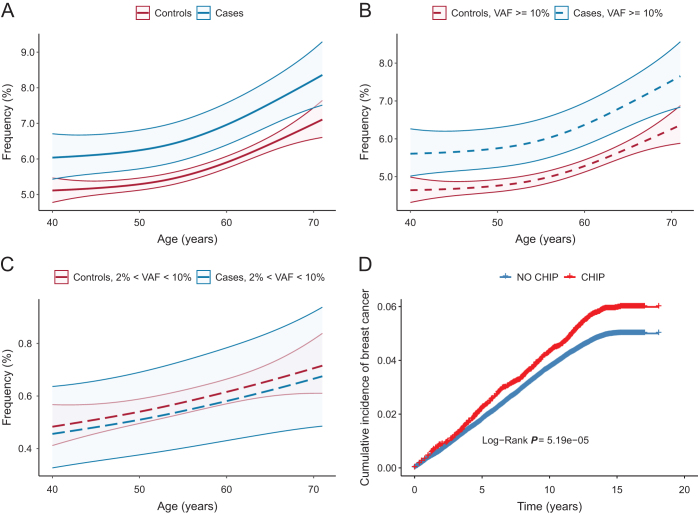
The prevalence of CHIP at baseline over age or time. (A). Frequency of overall CHIP in breast cancer cases and controls. (B) Frequency of CHIP high (VAF ≥ 10%) in breast cancer cases and controls. (C) Frequency of CHIP low (2% < VAF < 10%) in breast cancer cases and controls. (D). The cumulative incidence of breast cancer in population with CHIP or without CHIP over time.

Within the CHIP group, most individuals had high VAF (≥10%) or a single mutation (Table 1). The prevalence of CHIP was higher among breast cancer cases (6.79%) compared to controls (5.73%), primarily driven by those with high VAF (cases vs controls, 6.24% vs 5.14%). Using a generalized additive model with spline smoothing, we noted higher CHIP frequency in breast cancer cases across all ages (Fig. 2A–C). Cumulative incidence of breast cancer was also higher in participants with CHIP (Log-Rank *P* = 5.19e-05, Fig. 2D).

Univariable analysis revealed a significant association between CHIP and breast cancer in both logistic (odds ratio [OR] (95% confidence interval [CI]), 1.2001 [1.094–1.313], *P* = 8.94e-05) and CoxPH model (HR [95% CI], 1.2004 [1.099–1.312], *P* = 5.31e-05) (Table 2). Multivariable models adjusting for potential confounders (models a–c) confirmed a consistent 19% increased breast cancer risk associated with CHIP (Table 3). Analysis by VAF indicated an increasing trend in breast cancer risk (OR 0.93–1.22, *P*-trend = 6.51e-05).

**Table 2:** Association between CHIP and breast cancer assessed by univariable analysis based on logistic model and CoxPH model

Model	OR or HR	95% CI	*P* value
Logistic model	1.200124358	1.09426–1.31344	8.94e-05
CoxPH model	1.200454569	1.09867–1.31167	5.31e-05

CHIP, clonal hematopoiesis of indeterminate potential; CI, confidence interval; HR, hazard ratio; OR, odds ratio.

**Table 3:** Association between CHIP status and the risk of breast cancer assessed by multivariable analysis based on logistic model and CoxPH model

			VAF	
	CHIP carrier	2% to <10%	≥10%	*P* trend
No. cases (%)/controls (%)	525(6.8)/8502(5.7)	43(0.6)/869(0.6)	482(6.2)/7633(5.1)	
Logistic Model				
MV-adjusted OR (95% CI, *P*)^a^	1.19 (1.09–1.30, 1.62e-04)	0.95 (0.69–1.28, 0.746)	1.22 (1.11–1.34, 4.15e-05)	6.64e-05
MV-adjusted OR (95% CI, *P*)^b^	1.19 (1.09–1.30, 1.71e-04)	0.95 (0.69–1.27, 0.743)	1.22 (1.11–1.34, 4.34e-05)	6.99e-05
MV-adjusted OR (95% CI, *P*)^c^	1.19 (1.09–1.31, 1.77e-04)	0.93 (0.67–1.25, 0.645)	1.22 (1.11–1.35, 3.71e-05)	6.51e-05
CoxPH Model				
MV-adjusted HR (95% CI, *P*)^a^	1.19 (1.09–1.30, 1.09e-04)	0.95 (0.71–1.28, 0.747)	1.22 (1.11–1.34, 2.6e-05)	4.24e-05
MV-adjusted HR (95% CI, *P*)^b^	1.19 (1.09–1.30, 1.13e-04)	0.95 (0.70–1.28, 0.743)	1.22 (1.11–1.34, 2.68e-05)	4.41e-05
MV-adjusted HR (95% CI, *P*)^c^	1.19 (1.09v1.30, 1.24e-04)	0.93 (0.69–1.25, 0.62)	1.22 (1.11–1.34, 2.29e-05)	4.23e-05

BMI, body mass index; CHIP, clonal hematopoiesis of indeterminate potential; CKD, chronic kidney disease; CRP, C-reactive protein; CVD, cardiovascular disease; HR, hazards ratio; MV, multivariable; OR, odds ratio; PRS, polygenic risk score; SII, system inflammation index; VAF, variant allele frequency.

^a^ Adjusted by age, race, BMI, alcohol intake status, smoking status, diet quality score, physical activity, and assessment center.

^b^ Additionally adjusted by CRP, SII, history of diabetes, high cholesterol, hypertension, CKD, and CVD.

^c^ Additionally adjusted by the first 10 components of genetic ancestry and PRS of breast cancer. This model was used as the final model in other analysis.

## Stratified analysis on CHIP and breast cancer risk

**Figure 3.**
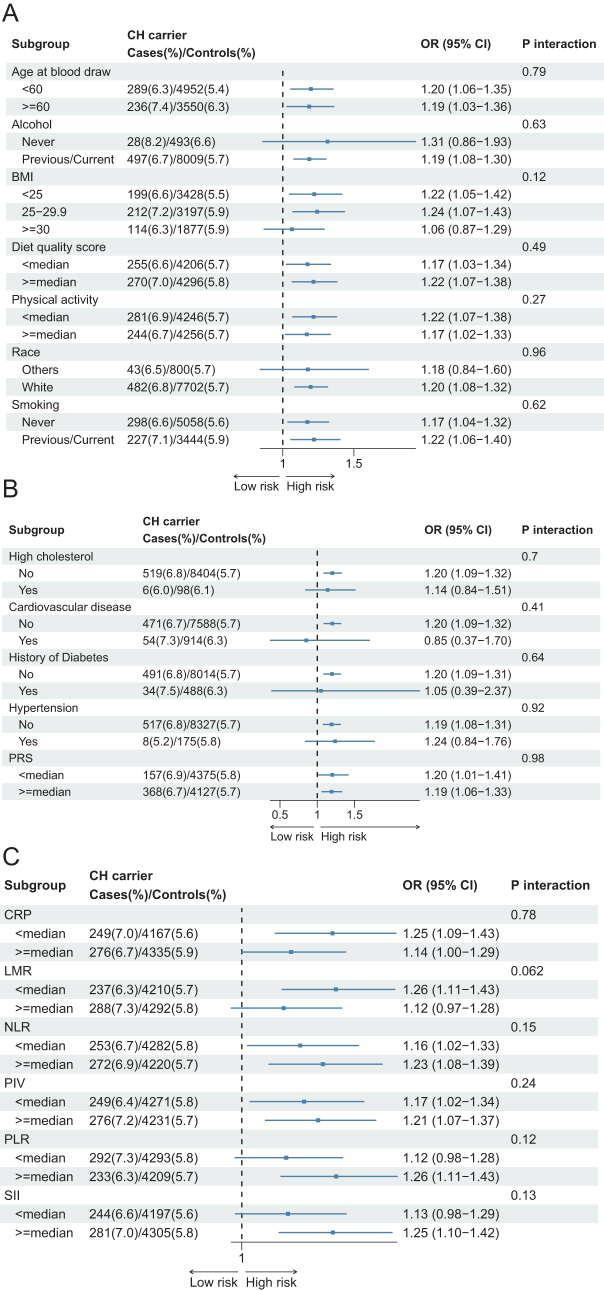
Forest plots showing the stratified analysis on the association between CHIP and risk of breast cancer. Participants were grouped into sub-groups based on potential risk factors: risk factors related to basic character or lifestyle (A), history of diseases or genetic factor (B) and inflammation status (C). In this figure, logistic model was used.

Stratified analyses across lifestyle factors, chronic disease history, and genetic risk factors consistently showed OR and HR > 1 for CHIP’s association with breast cancer, with no significant interactions (all *P* for interaction > 0.1, Fig. 3A-B and Supplementary Digital content, Figure 1A–B, Available at: http://links.lww.com/JS9/E845). An exception was found on LMR in inflammation-related stratification, where the *P* for interaction was 0.047 (CoxPH) and 0.062 (logistic) (Fig. 3C and Supplementary Digital content, SDC, Figure 1C, Available at: http://links.lww.com/JS9/E845). Sensitivity analyses excluding participants with extreme BMI or early follow-up periods (2 or 5 years) maintained the significant CHIP-breast cancer association (OR, 1.18; HR, 1.17, both *P* < 0.01, Table 4).

**Table 4:** Sensitivity analyses excluding participants under the first 2 or 5 years of follow-up and with extreme BMI

	Excluding by follow-up		Excluding by BMI
	First 2 years	First 5 years	<18.5 or >40 kg/m^2^
No. cases (%)/controls (%)	447(6.7)/8485(5.7)	327(6.7)/8409(5.7)	507(6.7)/8264(5.7)
Logistic model	1.18 (1.06–1.30)	1.18 (1.05–1.32)	1.18 (1.07–1.30)
(OR, CI, *P*)	1.38e-03	5.5e-03	4.84e-04
CoxPH model	1.17 (1.07–1.29)	1.17 (1.05–1.31)	1.18 (1.08v1.29)
(HR, CI, *P*)	1.04e-03	4.9e-03	3.42e-04

BMI, body mass index; CI, confidence interval; HR, hazards ratio; No., number of participants; OR, odds ratio.

### Traits of the CHIP–breast cancer relationship

**Figure 4.**
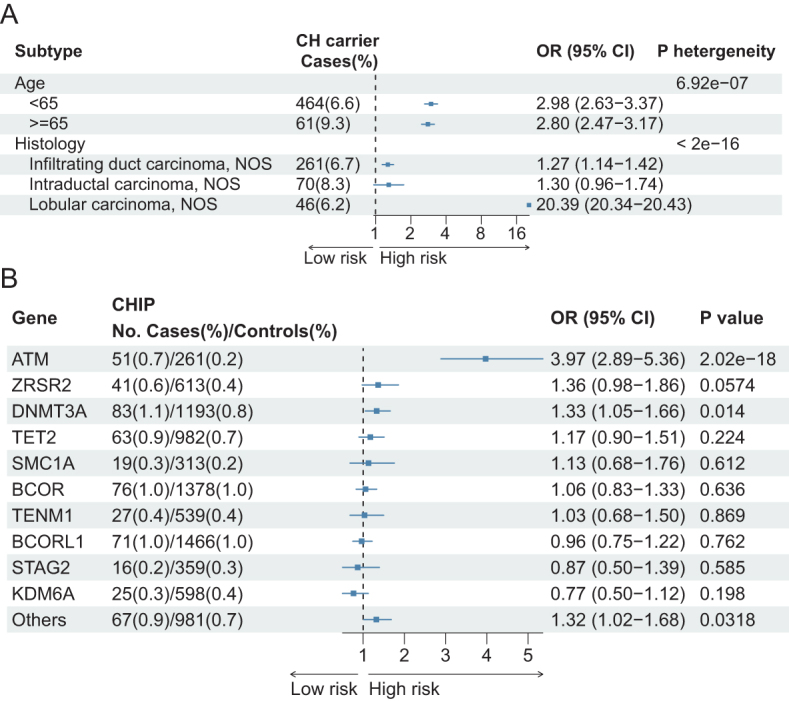
Forest plots show the different traits between the CHIP and the risk of breast cancer. (A). Participants were grouped by age when participants were diagnosed with breast cancer or by tumor histology. (B) Association between CHIP and breast cancer risk were examined at specific genes. Only the top 10 genes in population frequency were checked, other CHIP genes were summarized together. In this figure, logistic model was used.

Subgroup analysis based on breast cancer histology revealed a notably higher risk for “Lobular carcinoma, NOS” associated with CHIP (OR: 20.39, *P*-heterogeneity < 0.001) compared to “Infiltrating duct carcinoma” (OR: 1.27) and “Intraductal carcinoma, NOS” (OR: 1.30) (Fig. 4A). However, after performing the Fisher’s exact test, the high risk for “Lobular carcinoma, NOS” was absent (OR: 1.082, *P* = 0.58), which indicated that the OR with value 20.39 may be caused by its sparse data (only 746 cases) (Supplementary Digital content, SDC, Table 5, Available at: http://links.lww.com/JS9/E845). Furthermore, CHIP was linked to an older age at diagnosis (≥65 vs < 65 years: 9.3% vs 6.6%, *P*-heterogeneity < 0.001), though ORs were similar (2.98 vs 2.80).

**Figure 5.**
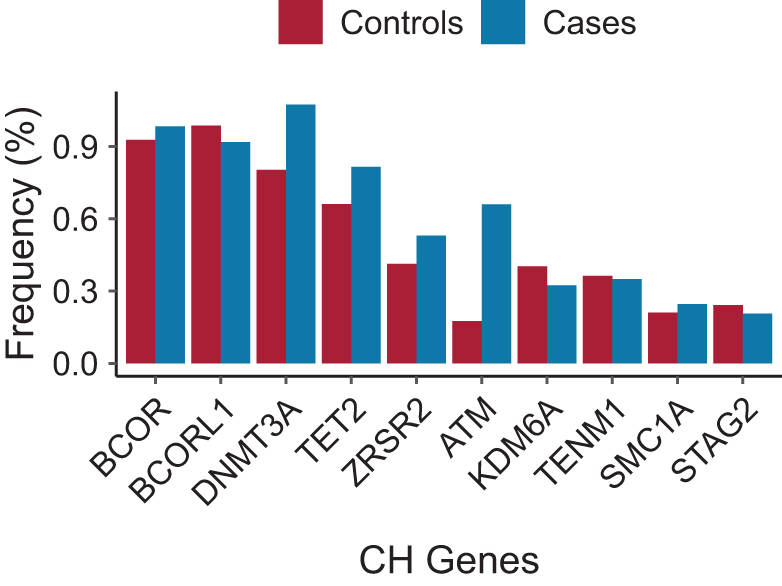
The bar plot indicates the different frequency of CHIP genes in control and breast cancer cases among the population.

We next investigated the effects of different CHIP mutated genes on breast cancer risk. Through the analysis of WES data, we identified the prevalence of the top 10 CHIP-associated gene mutations among individuals with CHIP. Our findings revealed that the genes *DNMT3A, TET2, ATM*, and *ZRSR2* exhibited a significant increase in frequency when comparing the case group to the control group (Fig. 5). Based on the logistic and CoxPH models, we discovered a significant association between the genes *ATM* (OR [95%CI], 3.97 [2.89–5.36], *P* = 2.02e-18) and *DNMT3A* (OR [95% CI], 1.33 [1.05–1.66], *P* = 0.014) and breast cancer risk (Fig. 4B and Supplementary Digital content, SDC, Figure 2, Available at: http://links.lww.com/JS9/E845). When grouping the non-top ten genes together, the association between CHIP and risk of breast cancer remained significant (OR [95% CI], 1.31 [1.03–1.67], *P* = 0.0318).

## Discussion

This prospective cohort study utilizing data from the UK Biobank highlights a significant association between CHIP and the increased risk of developing breast cancer. Our univariable analysis showed that CHIP may contribute to the risk of breast cancer. Importantly, this association remained significant even after adjusting for other known risk factors, such as age, smoking, and alcohol consumption. Further analysis of the ten most mutated CHIP genes revealed that mutations in *DNMT3A* and *ATM* were strongly linked to an elevated breast cancer risk. These findings suggest that CHIP may act as an independent risk factor for breast cancer and improve their early detection via genetic testing with the popularization of next-generation sequencing in clinical applications.

CHIP, while commonly regarded as a precursor state of hematologic neoplasms, it is also associated with dysregulated inflammatory responses and immune system disruptions, which may contribute to the development of a range of diseases, including cancer^[11,28]^. Most of the cells exhibiting CHIP mutations reside in the myeloid lineage, potentially compromising T cell functionality^[29]^. Mutations in TET2 in macrophages, for example, can increase inflammation via key inflammatory mediators such as interleukin-1β (IL-1β), IL-6, and IL-8^[30]^. Similarly, Dnmt3a has an important role in regulating responsiveness to acute or chronic stimulation in mast cells^[31]^, with deficiencies in DNMT3A potentially leading to ambient inflammation. Moreover, loss-of-function mutation in Dnmt3a and Tet2 may enhance inflammation in macrophages with the concordant phenotype^[32]^. Previous studies have identified specific CHIP mutations, such as those in TET2, JAK2 and AXSL1, as drivers of risk for diseases like acute kidney injury^[33]^ and lung cancer^[16]^. Notably, in a study involving hematopoietic-cell-transplanted mice, Tet2^-/-^ macrophages promoted liver inflammation, resulting in liver injury^[34]^. These findings underscore the variability of CHIP’s effects, which may differ depending on the context of the disease and the specific mutations involved^[35]^.

In our study, we found that mutations in DNMT3A and ATM were the most significant CHIP-associated mutations linked to breast cancer risk in both logistic and CoxPH models. ATM, a DNA damage response kinase involved in DNA repair, is known to play a role in immune cell functionality. Deficiency of ATM in T cells impairs their proliferation, which may hinder their capacity to undergo clonal expansion during activation^[36]^. Besides, ATM stabilizes DNA double-strand-break complexes in variable-diversity-joining rearrangement during T cell maturation and deficiency of ATM may result in a reduced diversity of TCR repertoire^[37]^. With reduced TCR repertoire and impaired activation, T cells may not effectively function to prevent the pathogenesis of breast cancer. DNMT3A mutation in myeloid lineages results in an ambience inflammation^[33–35]^, which contributes to the dysfunction of tumor microenvironment (TME) and cancer development^[38]^. In breast cancer, macrophages are an important component of the inflammatory infiltrate which shapes an immune suppressive TME^[39]^. In a study on the association between CHIP and lung cancer^[16]^, Tian *et al* found the most prevalent CHIP genes related to lung cancer were ASXL1 (OR [95% CI], 2.00 [1.02–3.91]), DNMT3A (1.29 [0.94–1.76]), and TET2 (1.31 [0.63–2.75]). DNMT3A were found to be the common risk driver gene in both studies. Unlike our study, they found that mutations in ASXL1 exhibited a higher risk of lung cancer than DNMT3A and TET2. This phenomenon could be explained by smoking, which is a well-known high-risk factor of lung cancer and is highly related to mutations in ASXL1^[40]^. Taken together, CHIP may play both correlational and causal role in breast cancer pathogenesis. However, further experimental studies are needed to elucidate the underlying mechanisms through which CHIP contributes to breast cancer and clarify the role of CHIP in breast cancer.

Our study has several strengths. First, the large-scale, population-based cohort enriched with detailed genomic and phenotypic data enabled us to conduct a robust prospective analysis of the relationship between CHIP and breast cancer risk. Second, we made extensive adjustments for potential confounders using three different multivariable models. Third, sensitivity analyses, including stratification, were performed to further validate the reliability of our findings. Fourth, we accounted for the potential influence of extreme values by excluding outlier participants, ensuring a more accurate analysis. Finally, the use of both logistic and CoxPH models allowed us to cross-validate our results, providing greater confidence in the robustness of our findings.

However, our study also has some limitations. Despite the large sample size, the findings would be more convincing if they were validated in independent cohorts. Additionally, the precise biological mechanisms underlying the association between CHIP and breast cancer risk remain to be fully explored. The UK Biobank’s phenotypic data were only collected at baseline, limiting our ability to assess changes in participant characteristics over time, especially the CHIP progression. Longitudinal studies are crucial for describing how CHIP changes over time including their growth rates and factors influencing their expansion or shrinkage. Moreover, the exclusion of individuals may lead to selection bias, which could influence the generalizability and representativeness of current results. Furthermore, while we applied various analytical strategies, unmeasured confounders inherent to observational studies may still influence the results, for example, the abnormal immune system function may affect the pathogenesis of disease, future study should assess such confounders and include them in sensitive analysis. Finally, we could not explore the correlation between CHIP and different breast cancer stages because of a lack of clinical information. Aimed at addressing the limitations of our study, further research should include validating current findings in independent cohorts, such as The Cancer Genome Atlas. This validation step is essential to make current results more robust and general across different populations and datasets. Additionally, exploring the interplay between CHIP, immune system and breast cancer in vitro will provide critical clues for uncovering the potential underlying mechanisms.

## Conclusion

In conclusion, our study demonstrates that CHIP is associated with an increased risk of developing breast cancer. These findings suggest that CHIP could potentially serve as a biomarker for identifying individuals at higher risk for early-stage breast cancer detection. However, further research is necessary to validate these results and explore the underlying mechanisms.

## Data Availability

This work was performed on the UK Biobank with application number 98937. Due to the privacy and sensitive information contained in the data, data used will not be publicly available. The raw and derived data from UK Biobank (https://www.ukbiobank.ac.uk/) are available to approved researchers.

## Research registration unique identifying number (UIN)

Our study is a retrospective analysis based on data from a public database UK Biobank. As this study involves the use of de-identified, publicly available data and does not involve direct intervention with human subjects, it is exempt from registration.
